# Stage-specific threats reveal the inadequacy of adult-centered conservation

**DOI:** 10.7554/eLife.110823

**Published:** 2026-09-07

**Authors:** Yan-Fang Song, Yong-Le Wang, Qing-Qing Li, Zhi-Yong Yuan, Wei-Wei Zhou

**Affiliations:** 1 Key Laboratory for Conserving Wildlife with Small Populations in Yunnan, Forestry College, Southwest Forestry University Kunming China; 2 State Key Laboratory of Herbage Improvement and Grassland Agro-ecosystems, and College of Ecology, Lanzhou University Lanzhou China; 3 Key Laboratory of Freshwater Fish Reproduction and Development (Ministry of Education), School of Life Sciences, Southwest University Chongqing China; University of Zurich Switzerland; Max Planck Institute for Biology Tübingen Germany

**Keywords:** complex life cycles, anura, extinction risk, amphibian conservation, functional trait, Other

## Abstract

In an era of severe global biodiversity threats, understanding the link between species’ traits and their endangerment helps uncover causes of risk and infer threats to understudied species. Most animals have complex life cycles with distinct stages that may face stage-specific threats. Current conservation frameworks rely heavily on adult traits, potentially misjudging extinction risk. Using Chinese anurans as a model, we integrated functional traits from both adult and tadpole stages to examine their association with extinction risk. We found that body size positively correlates with risk in both stages. Microhabitat use is related to extinction risk in tadpoles, but shows no significant link in adults. Adult relative tympanum diameter and head length also correlate with extinction risk. These results indicate that species vulnerability is correlated with multi-stage traits, with both shared and stage-specific threats. Conservation based solely on adult traits may fail to accurately assess species threats. We call for integrating a whole-life-history perspective into biodiversity assessment and conservation to more effectively address the global biodiversity crisis.

## eLife digest

Biodiversity is declining, and many species may face extinction in the future. Some species are more vulnerable to human-driven threats than others, but limited resources make it impossible to assess the extinction risk of every species in detail. Certain traits, such as body size, are known to be associated with extinction risk, so understanding these relationships can help predict which species may be most threatened.

However, many animal species have complex life cycles. Frogs, beetles and butterflies, for example, undergo dramatic changes in body form, behaviour and habitat as they develop from larvae to adults. Yet conservation assessments focus almost exclusively on adult traits, largely overlooking the distinctive threats faced by younger life stages. This could lead to underestimating a species’ overall extinction risk.

Song et al. asked whether traits of both adults and larvae are associated with extinction risk and, if so, whether these relationships are consistent across life stages. This is important because if threats differ between stages, an adult-centred approach could overlook vulnerabilities earlier in life. For amphibians, for example, protecting adult habitats while neglecting the specific needs of tadpoles could lead to ineffective conservation strategies.

Studying nearly 300 Chinese amphibian species, Song et al. found that extinction risk was associated with traits from both adult and tadpole stages. Some associations were shared across life stages: larger body size was linked to higher extinction risk in both tadpoles and adults. They also identified stage-specific associations. Habitats used by tadpoles were related to extinction risk, whereas they did not find the same relationship for adult habitat.

Because most described animal species, including many insects, crustaceans and other invertebrates, have complex life cycles, comparing trait-threat relationships across life stages could be widely applicable. The findings of Song et al. highlight the importance of considering vulnerabilities at all stages of the life cycle when assessing extinction risk and developing conservation strategies.

To make conservation planning more effective, we need better data on the functional traits and ecological requirements of non-adult life stages, formal integration of information from multiple life stages into conservation assessments, and habitat protection that accounts for the needs of all life stages, particularly habitats that are critical for larvae.

## Introduction

Human activities are driving global biodiversity loss at an unprecedented rate and scale, placing a vast number of species at risk of extinction (Cowie et al., 2022; Keck et al., 2025). However, not all species are affected equally or in the same manner. Species with different ecological strategies exhibit marked differences in vulnerability when facing the anthropogenic disturbances (Chichorro et al., 2022). Although the statistical associations between traits and extinction risk may not imply causation, investigating the relationship between species’ traits and extinction risk is of great importance for revealing causes of endangerment and predicting potential threats. On one hand, understanding the relationship between traits and extinction risk helps identify species’ characteristics that may make it vulnerable, thereby clarifying the causes of their endangerment. For example, body size, as the most fundamental and commonly measured ecological trait, has been shown to be closely related to extinction risk across numerous taxa (Tomiya, 2013; Vilela et al., 2014; Nolte et al., 2019; Cardillo, 2021). The relationships between body sizes and extinction risks vary among different groups. Large species often face higher risks due to low reproductive rates, narrow distributions, or greater susceptibility to human hunting (Keane et al., 2005; Ruland and Jeschke, 2017). But in some taxa, small species may be more prone to extinction due to smaller geographic ranges, weak dispersal ability, or habitat specialization (Owens and Bennett, 2000; Cardillo, 2021). On the other hand, our knowledge of biodiversity remains incomplete. Even for vertebrate groups, a large number of new species are described annually (Uetz et al., 2025; Frost, 2026). Existing assessment systems struggle to promptly evaluate the extinction risk of these new species. Understanding the relationship between traits and extinction risk can provide a basis for risk prediction for species that are not yet assessed or are data-deficient (González-del-Pliego et al., 2019).

Exploring the relationship between species’ functional traits and extinction risk is very important for biodiversity conservation (Chichorro et al., 2019). However, a long-overlooked issue is that over 80% of described animal species have complex life cycles (Werner, 1988; Phung et al., 2020). In these groups, different life stages are often highly differentiated in morphology, physiology, behavior, and habitat requirements (Moran, 1994). According to the adaptive decoupling hypothesis, trait evolution in these stages often faces different selective pressures and shows relative independence (Wollenberg Valero et al., 2017). This hypothesis has been supported in many groups (Phillips, 1998; Sherratt et al., 2017; Goedert and Calsbeek, 2019). It follows that the threats and vulnerabilities a species faces at different stages may also differ. Unfortunately, studies on drivers of extinction risk always rely on adult traits, with minimal attention paid to juvenile or non-adult stages, leading to potential systematic bias in our understanding of a species’ overall vulnerability. For instance, in amphibians, which is a typical group with biphasic life cycles, conservation measures that neglect larval stages are often less effective (Nolan et al., 2023). Furthermore, studies have shown that biodiversity hotspots for tadpoles and adults do not overlap, and conservation priority species defined based on a single stage may have great biases in amphibians (Song et al., 2025a). Meanwhile, incorporating traits from life stages other than adults into models allows for the development of more robust statistical associations between traits and threat status, thereby enhancing the effectiveness of biodiversity conservation efforts.

Anuran amphibians serve as an ideal model system for investigating how functional traits across different life stages relate to extinction risk (Phung et al., 2020). As a group characterized by complex life cycles, amphibians exhibit striking differences between adults and their larval stage, also known as tadpoles, in morphology, diet, microhabitat selection, and behavior (McDiarmid and Altig, 1999). Furthermore, with over 40% of amphibian species facing extinction risk, they rank among the most threatened vertebrate groups (IUCN, 2025). While population models suggest that survival during the tadpole stage may not be the most critical determinant of overall population persistence (Biek et al., 2002), and other studies have highlighted the importance of the post-metamorphic juvenile stage (Petrovan and Schmidt, 2019), the tadpole stage nevertheless remains a vulnerable phase in amphibian development, characterized by high mortality and sensitivity to disturbances (Nolan et al., 2023). Consequently, insufficient understanding of this life stage may hinder effective conservation of the entire group (Nolan et al., 2023). Examining the relationship between functional traits at different life stages and extinction risk in amphibians not only aids in protecting this unique and endangered group but also offers valuable insights for other taxa with complex life histories. Regrettably, most existing research has focused primarily on adult traits (Cooper et al., 2008; Chen et al., 2019; Cardillo, 2021). Although some studies incorporate reproductive traits, the link between tadpole-stage traits and extinction risk remains poorly understood (Chen et al., 2019). At the same time, although tadpole conservation has received attention in some amphibian conservation efforts (Calhoun et al., 2014; Moor et al., 2022; Vredenburg, 2004), current conservation assessment frameworks and conservation planning still generally lack the integration of information on this life stage, which may reduce the effectiveness of these conservation measures. This limitation observed in amphibians may be widespread across most animal groups with complex life cycles (Faria et al., 2021). Therefore, using amphibians as a model, the research framework considering information from different life history stages holds broad implications for advancing the conservation of complex life cycle taxa throughout the animal kingdom. It helps address the bias in current conservation programs, which are heavily relying on information of adults.

For groups with complex life histories, the lack of data on stages other than adults severely limits the related research. China harbors exceptional amphibian diversity and has accumulated comprehensive, systematic data on species taxonomy, distribution, ecology, and conservation status (Fei et al., 2009a; Fei et al., 2009b; Fei, 2020; Huang et al., 2023; Song et al., 2025a; Song et al., 2025b; Wei et al., 2026; AmphibiaChina, 2026), providing a unique opportunity for integrated analyses of multi-stage traits and extinction risk. Critically, these data include high-resolution continuous functional trait data, which, compared to traditional discrete or categorical variables, can more finely depict variation in species’ ecological strategies (Tobias et al., 2022). Based on high-quality data, we will use Chinese anuran species to address two questions by studying the relationship between extinction risk and functional traits of adults and tadpoles: (1) Are functional traits of both adults and tadpoles correlated with species extinction risk? (2) If correlated, are these associations consistent across different life stages? By comparing correlations between traits and extinction risk across different stages, we aim to evaluate whether the adult-based conservation assessment system adequately reflects the vulnerability of species with complex life histories.

## Results

### Threat status of Chinese anurans

**Figure 1.**
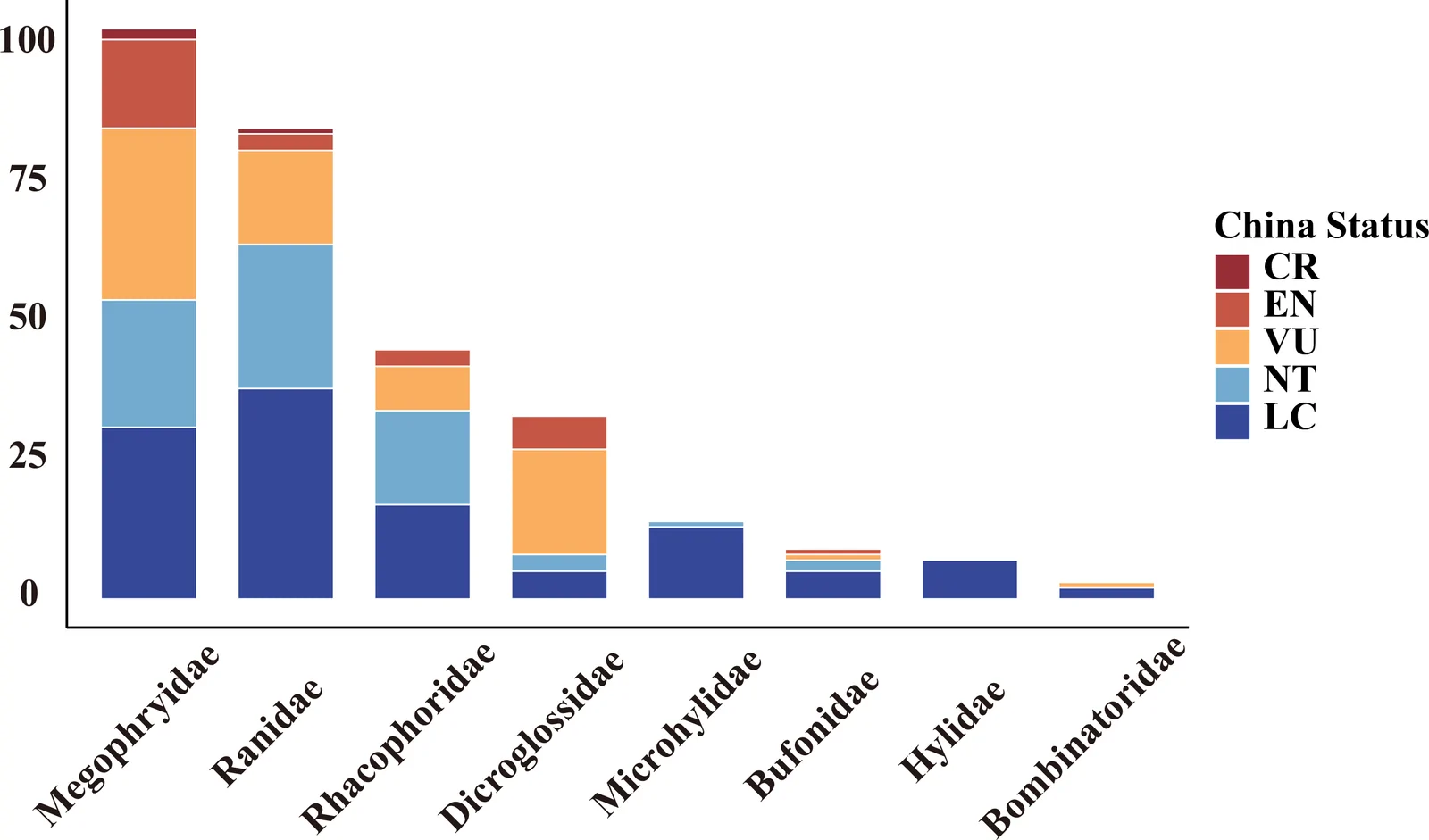
Distribution of threat status across anuran families in China, based on the China Biodiversity Red List – Vertebrates Volume (2020). Each stacked bar represents the proportion of species within a family categorized as Critically Endangered (CR), Endangered (EN), Vulnerable (VU), Near Threatened (NT), or Least Concern (LC). Families are ordered by total number of species.

After excluding species not evaluated or data deficient in the China Biodiversity Red List, 299 species were included in the analysis. Threat status varied markedly among families (Figure 1). Dicroglossidae exhibited the highest level of threat: 58% of species were classified as Vulnerable (VU) and 18% as Endangered (EN). Megophryidae also faced high threats, with nearly half (48%) of species in threatened categories. A total of 2% of species were classified as Critically Endangered, 30% as VU, and 16% as EN. In Bufonidae, Ranidae, and Rhacophoridae, the proportion of threatened species was 22%, 25%, and 24%, respectively. In contrast, Hylidae and Microhylidae consisted almost entirely of Least Concern (LC) species, with no threatened taxa recorded. Bombinatoridae had no CR or EN species, but 33% of species were listed as VU. The results of the Spearman test support a significant correlation between the threat categories assigned in the China Biodiversity Red List and those in the International Union for Conservation of Nature (IUCN) Red List (Spearman’s ρ=0.573, p<0.001, n=257).

**Figure 1—figure supplement 1.**
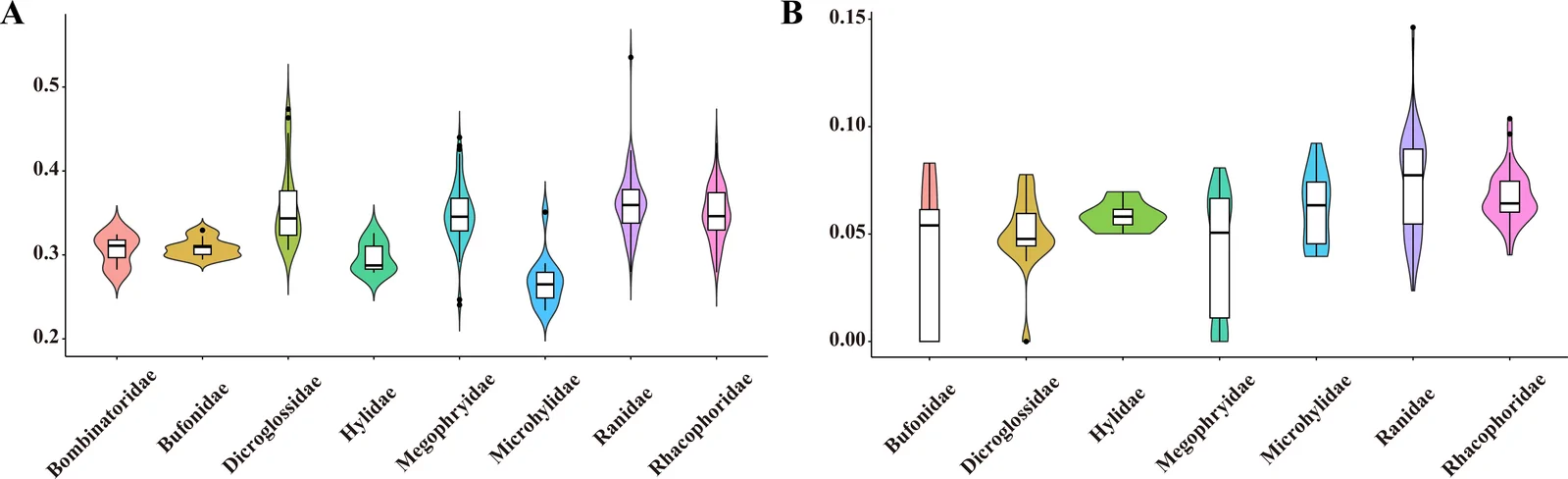
Violin plots showing the distribution of (**A**) head length and (**B**) tympanum diameter across anuran families in China. Species of the Bombinatoridae lack a tympanum; therefore, they are marked as 0 and are not shown in B. Additionally, because other families also include species without a tympanum, the kernel density estimation produces boundary effects, leading to negative values in the plots. Thus, we truncated the density estimation at the data boundaries during visualization. Each violin plot represents the kernel density estimate of trait values, with the inner boxplot indicating median, interquartile range, and outliers. Traits are standardized relative to snout-vent length.

**Figure 1—figure supplement 2.**
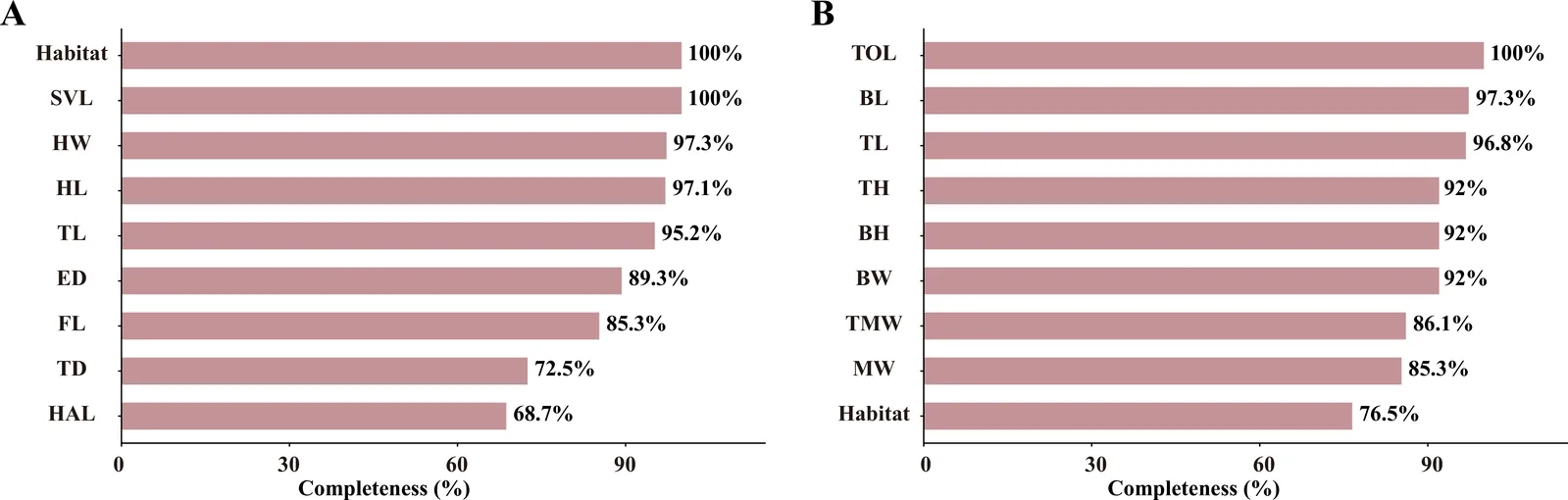
Data completeness of each trait for (**A**) adults and (**B**) tadpoles.

### Extinction risk correlates in adults and tadpoles

**Figure 2.**
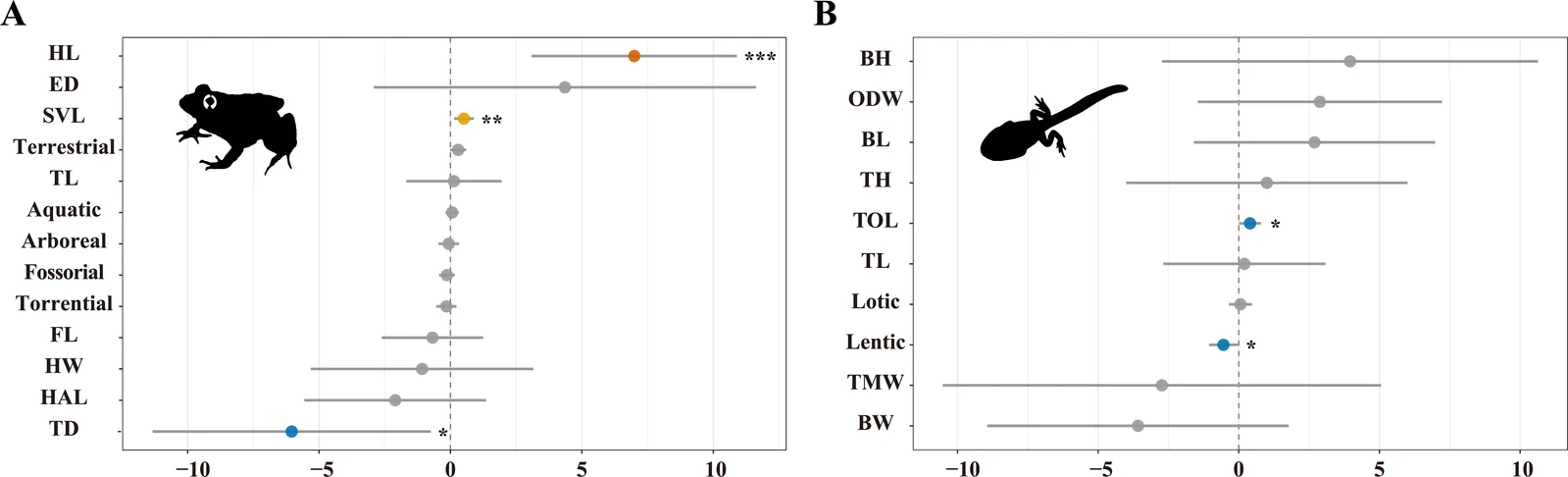
Phylogenetically corrected associations between functional traits and extinction risk in Chinese anurans, separately for (**A**) adults and (**B**) tadpoles. Each dot represents the phylogenetic generalized least squares (PGLS) regression coefficient (β) for a given trait, with horizontal lines indicating 95% confidence intervals. Traits significantly associated with threat status are marked with asterisks (*p<0.05; **p<0.01; ***p<0.001). For abbreviations of traits, please refer to Table 3. The silhouette images are sourced from phylopic (*Minervarya mudduraja* by Vijay Karthick; *Bufo bufo* by Luca Leicht).

Model comparisons revealed that the OU-based model provided the best fit for both adult and tadpole datasets (Table 1). In adults, snout-vent length, head length, and tympanum diameter showed significant associations with threat status (Figure 2A). Specifically, snout-vent length (β=0.52, p<0.01) and head length (β=6.86, p<0.001) were positively correlated with threat level, whereas tympanum diameter (β = –5.91, p<0.05) was negatively correlated. Other morphological variables such as head width, eye diameter, and traits about forelimb and hindlimb showed no significant effects. Notably, adult microhabitat types were not significantly associated with extinction risk. Furthermore, the analysis based on model averaging yielded similar results (Figure 2—figure supplement 1). HL, SVL, and TD were the three most important variables to explain extinction risk.

**Figure 2—figure supplement 1.**
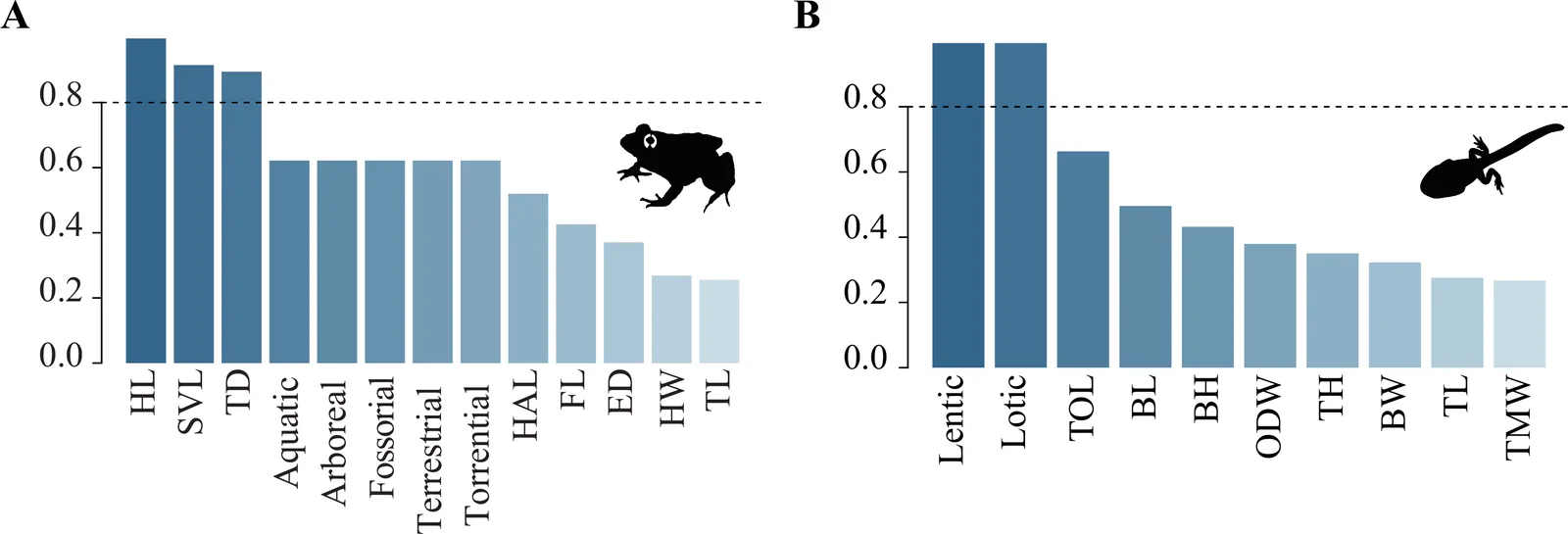
Trait importance (sum of Akaike weights) of adult and tadpole traits based on model averaging under the Ornstein-Uhlenbeck (OU) evolutionary model using China Biodiversity Red List. For abbreviations of traits, please refer to Table 3. The silhouette images are sourced from phylopic (*Minervarya mudduraja* by Vijay Karthick; *Bufo bufo* by Luca Leicht).

**Figure 2—figure supplement 2.**
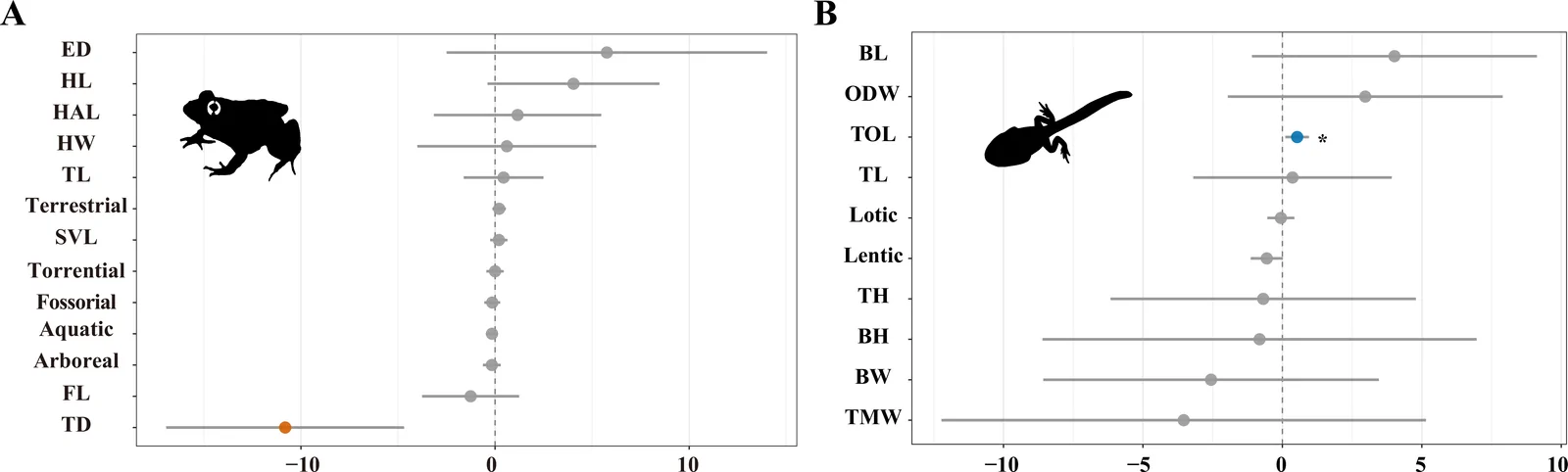
Phylogenetically corrected associations between functional traits and extinction risk in Chinese anurans based on International Union for Conservation of Nature (IUCN) Red List for (**A**) adults and (**B**) tadpoles. Each dot represents the phylogenetic generalized least squares (PGLS) regression coefficient (β) for a given trait, with horizontal lines indicating 95% confidence intervals. Traits significantly associated with threat status are marked with asterisks (*p<0.05; **p<0.01; ***p<0.001). For abbreviations of traits, please refer to Table 3. The silhouette images are sourced from phylopic (*Minervarya mudduraja* by Vijay Karthick; *Bufo bufo* by Luca Leicht).

**Figure 2—figure supplement 3.**
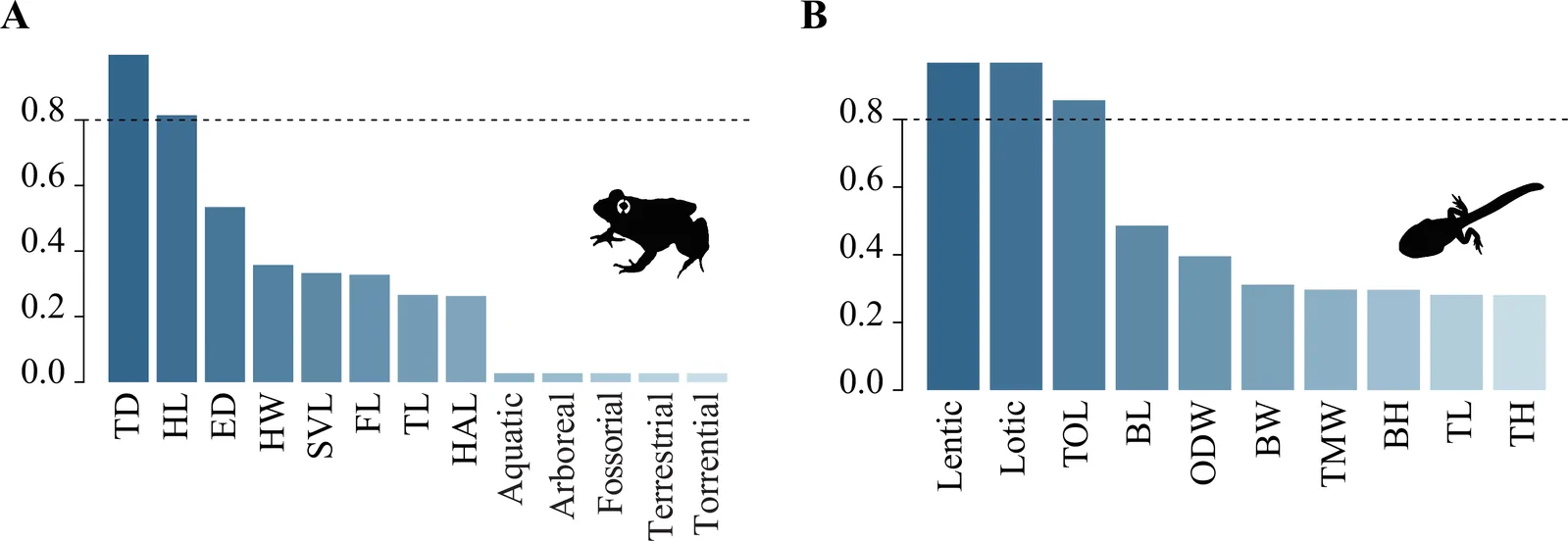
Trait importance (sum of Akaike weights) of adult and tadpole traits based on model averaging under the Ornstein-Uhlenbeck (OU) evolutionary model using International Union for Conservation of Nature (IUCN) Red List. For abbreviations of traits, please refer to Table 3. The silhouette images are sourced from phylopic (*Minervarya mudduraja* by Vijay Karthick; *Bufo bufo* by Luca Leicht).

**Table 1.** Regression analysis results of extinction risk for adult and tadpole stages. For abbreviations of traits, please refer to Table 3. BM, Brownian motion; OU, Ornstein-Uhlenbeck; GLS, generalized least squares; PGLS, phylogenetic generalized least squares; AICc, Akaike information criterion corrected for small sample size.

Life stage	Model type	AICc	Model
Adult	PGLS (BM)	1028.36	/
	PGLS (OU)	859.95	China extinction risk ~ 0.52 SVL** + 6.86 HL*** – 0.86 HW + 4.30 ED – 5.91 TD* – 2.06 HAL – 0.68 FL + 0.17 TL – 0.07 Arboreal – 0.13 Fossorial + 0.28 Terrestrial + 0.07 Aquatic – 0.17 Torrential
	GLS (non-phylogenetic)	861.90	/
Tadpole	PGLS (BM)	1032.56	/
	PGLS (OU)	874.03	China extinction risk ~ 0.40 TOL* + 2.69 BL – 3.58 BW + 3.96 BH + 0.20 TL + 1.00 TH – 2.73 TMW + 2.88 MW – 0.55 Lentic* + 0.06 Lotic
	GLS (non-phylogenetic)	876.92	/

*p < 0.05, **p < 0.01, ***p < 0.001.

In tadpoles, only total length and lentic microhabitat use showed significant associations (Figure 2B). Similar to adults, tadpole body size had a significant positive effect (β=0.40, p<0.05). Lentic habitat type showed a significant negative correlation (β=–0.55, p<0.05), indicating that species which have tadpoles living in lentic environments had relatively lower extinction risk. None of the other traits showed significant correlations. In the model averaging analysis, the two variables representing habitat were the most important, and the third most important was total length, which represents body size. Correlation between adult and tadpole body size was very weak across all species (r=0.12), with variation among families (Table 2). In the analysis based on IUCN Red List data, the regression coefficients for each trait were similar, but some traits’ coefficients were not significant, including adult head length, body size, and tadpole microhabitat type (Figure 2—figure supplements 2 and 3, Supplementary file 1A).

**Table 2.** Relationships between tadpole and adult size within anuran families. SVL: snout-vent length; TOL: total length; d: parameter quantifying the rate of return to the optimal value in the Ornstein-Uhlenbeck (OU) evolutionary model, reflecting the strength of phylogenetic signal.

Family	Coefficient	d (SVL)	d (TOL)
All	0.12	0.58	0.13
Bufonidae	0.11	0.00	2.74
Dicroglossidae	0.30	0.79	0.06
Hylidae	–0.12	0.22	1.47
Megophryidae	0.33	0.67	0.09
Microhylidae	0.37	1.93	0.53
Ranidae	0.34	0.00	0.00
Rhacophoridae	0.24	0.50	0.55

## Discussion

By integrating functional trait data from both adult and tadpole stages, we reveal statistical associations between multi-stage traits and extinction risk in anurans. The results support that species’ threat status is related to traits from both life history stages, and the nature of these associations can be different between stages. Given that over 80% of described animal species have complex life histories (Werner, 1988; Phung et al., 2020), and current conservation programs heavily rely on adult information (Faria et al., 2021), our study suggests that neglecting the vulnerability of non-adult stages may lead to a systematic underestimation of a species’ overall extinction risk. Therefore, to better protect biodiversity, multi-stage perspectives must be incorporated into assessment frameworks and conservation planning (Song et al., 2025a; Roehrdanz, 2026).

### Differences in threats across life stages

Our results confirm that different life stages face distinct threats. Our analysis clearly showed that tadpole microhabitat type was significantly correlated with extinction risk, while adult microhabitat type showed no such association. Although the results of the model-averaging analysis slightly differed from those of the full model, both supported that tadpole microhabitat type is associated with extinction risk. Lotic microhabitat was also an important variable explaining extinction risk in the model-averaging analysis. Although it was not significant in the full model, the regression coefficient between lotic microhabitat and extinction risk was positive. All the evidence supports species with tadpoles living in lentic environments (e.g. ponds) faced significantly lower extinction risk. Habitat alteration caused by human activities is a major threat to biodiversity (Keck et al., 2025). Our results indicate that the impact of habitat change differs across life stages. Therefore, focusing only on one stage, such as adults, may underestimate the threats to biodiversity. Tadpoles are more dependent on aquatic environments than adults (McDiarmid and Altig, 1999), making them particularly sensitive to waterbody degradation (Nolan et al., 2023). The significantly lower extinction threat for lentic tadpoles may be because human-induced habitat changes have a lesser impact on lentic habitats. For example, human-dominated environments, including cities and farmlands, are more likely to retain lentic water bodies, such as ponds in urban parks or farmland (Hamer and Parris, 2011; Holzer, 2014). This result suggests that in amphibian conservation, more attention should be paid to protecting larval habitats. We also recommend that tadpole microhabitat sensitivity be considered as an auxiliary indicator in future species extinction risk assessments.

Apart from body size and microhabitats, no other functional traits in tadpoles showed a significant relationship with extinction risk. However, for adults, besides body size, relative tympanum size and head length were also significantly correlated with extinction risk. We found that species with relatively smaller tympanum faced higher extinction risk. Tympanum size is related to acoustic communication in amphibians, and most anurans rely heavily on acoustic communication for reproduction (Schwartz et al., 2001). Studies have shown that anthropogenic noise causes auditory masking in frogs and reduces individual reproductive success (Caorsi et al., 2019; Grenat et al., 2019; Grenat et al., 2024). It also alters community-level acoustic characteristics (Zhao et al., 2025a). However, this evidence is largely limited to behavioral changes, and no study has directly linked noise to amphibian population viability. Additionally, the IUCN does not recognize noise as a major threat to amphibians. Thus, the link between tympanum size and extinction risk is proposed as a testable hypothesis, and the causal mechanisms need future verification.

We also detected a significant positive correlation between relative head length and extinction risk. Species with larger relative head lengths had greater extinction risk. Skull morphology is related to both habitat selection and diets in anurans (Paluh et al., 2020). For example, fossorial species tend to have short, high skulls, while aquatic species often possess elongated, flattened heads. Unfortunately, for the species involved in our study, research on the threat differences faced by species with different relative head lengths is still lacking. Simultaneously, we found that relative head length varied greatly among different families (Figure 1—figure supplement 1). In several families with high species richness and many endangered species, relative head length was larger, such as in Megophryidae and Dicroglossidae. Therefore, we cannot completely rule out the possibility that the relationship between relative head length and extinction risk is because species in these families have larger relative head lengths. Although we corrected for the influence of phylogenetic relationships in the analysis, strong coupling between taxonomic group characteristics and threat status may still leave a signal. Although the underlying mechanism remains unclear, which warrants further investigation, our study discovered a potential link between adult head length and extinction risk.

Our results clearly indicate that threats differ between adult and tadpole stages in amphibians. These differences suggest that characteristics of different life stages should receive attention in biodiversity conservation. For example, the impact of various human activities on tadpole habitats should receive more focus. Furthermore, it is highly likely that such differences exist in other groups with complex life cycles. Although the vast majority of described animal species have complex life histories, we have a widespread knowledge gap regarding all aspects of stages other than adults (Faria et al., 2021; Nori et al., 2025). This gap may greatly hinder biodiversity conservation policy-making and related research.

### Common threats across life stages

Despite stage differences in threats, we also identified a common association throughout the life cycle: larger body size is associated with higher extinction risk. Significant positive correlations were observed in both adults and tadpoles. Among various functional traits, body size is the most commonly associated functional trait with extinction risks (Tomiya, 2013; Seibold et al., 2015; Terzopoulou et al., 2015; Verde Arregoitia, 2016). Several mechanisms may explain this pattern. Large species are often preferentially targeted by humans for consumption or trade (Terzopoulou et al., 2015; Verde Arregoitia, 2016; Chichorro et al., 2019). In China, large frogs such as *Quasipaa spinosa*, *Hoplobatrachus chinensis*, and *Rana dybowskii* have suffered severe population declines due to overharvesting (Fei et al., 2009b; Tian et al., 2011; Chan et al., 2014; Zhao et al., 2014). Our results suggest that human harvesting may also threaten species at the tadpole stage. Although less common than adults, in many regions, tadpoles are also collected for food or traditional medicine (Luo et al., 2023; Liu et al., 2024). Furthermore, smaller-bodied species typically exhibit higher natural abundance (Damuth, 1981), which may provide them with a certain buffer against extinction risk. Alternatively, as body size is related to a species’ resource acquisition, larger species may require more space or resources (Purvis et al., 2000; Chichorro et al., 2019). When the environment is changed or habitat becomes fragmented, difficulties in resource accessibility may also cause species endangerment (Womersley et al., 2024; Ning et al., 2025; Zhao et al., 2025b). This could also be a reason for the positive correlation between body size and extinction risk. Regardless of the exact mechanism, the consistent positive association between size and risk has practical utility. In the absence of detailed life history data, body size can serve as a proxy variable for preliminary risk screening for Chinese amphibians. In extinction risk assessments or conservation priority ranking, species with larger body sizes, whether adults or tadpoles, should receive more attention.

Of course, we also cannot exclude another hypothesis, which is that adult and tadpole body sizes are correlated. Previous studies found a weak correlation between adult and tadpole body sizes, but this correlation varies among different groups (Phung et al., 2020). Among the species involved in this study, adult and larval body sizes showed only a very weak correlation, with a correlation coefficient of only 0.12 (Table 2). Therefore, although we cannot completely rule out the possibility that the relationship between tadpole body size and extinction risk is due to a correlation between tadpole and adult body sizes, our study supports a conservative inference: species possessing larger tadpoles overall face higher risk, and this risk should not be neglected in conservation planning.

## Methods

### Data collection

Extinction risk data were sourced from the China Biodiversity Red List: Vertebrates Volume (2020) (The Ministry of Environment Protection and The Chinese Academy of Sciences, 2023), which is the latest species threat category list for species in China currently. Compared to the IUCN Red List, this list is updated more timely for Chinese species and additionally covers the threat categories of 42 species. Similar to the IUCN Red List, the China Biodiversity Red List categories are assigned based on population size and distribution range, and are independent of species’ traits. We also assessed the similarity of species assessments between the two lists based on Spearman correlation and reanalyzed the data based on the IUCN Red List. Since our aim was to examine relationships between traits and threat status, rather than predicting the threat status of unevaluated species, we excluded species that were data-deficient. Following the method of previous studies (Cardillo, 2021), we performed an ordered coding conversion according to the severity of species threat categories: Least Concern (LC)=0; Near Threatened (NT)=1; Vulnerable (VU)=2; Endangered (EN)=3; Critically Endangered (CR)=4.

Functional traits can reflect species’ interactions with the environment (Violle et al., 2007). We selected eight morphological traits of both adults and tadpoles related to locomotion, foraging, and ecological habits from functional trait databases (Huang et al., 2023; Song et al., 2025b). These traits are closely related to the ecological function and performance of species (Table 3). Tadpole traits were collected specifically during Gosner stages 32–40, when taxonomically diagnostic characters remain stable (Haas et al., 2022). Except for body size, all other morphological data were converted to ratios relative to total length of tadpoles or snout-vent length of adults. In addition to morphological traits, previous studies have shown that microhabitat type is a strong predictor of extinction risk (Seibold et al., 2015). Therefore, we also included microhabitat data for both adults and tadpoles, encoded as dummy variables. Although amphibians typically breed in aquatic environments, most species spend most of their lives outside water. Thus, microhabitat here refers to the primary non-breeding habitat. Adult microhabitats were classified as: arboreal, fossorial, terrestrial, aquatic, and torrential. Tadpole microhabitats were categorized as lentic or lotic. If a species can inhabit multiple microhabitats, all applicable types were annotated. For example, semi-aquatic was coded as being able to inhabit both terrestrial and aquatic habitats. As our purpose was to compare the relationships between adults’ and tadpoles’ traits and the extinction risk, we did not include traits that have been shown to be closely associated with extinction risk, such as geographic range, elevational range, fecundity, and habitat specificity.

**Table 3.** Functional traits and their potential ecological functions.

Life stage	Functional trait	Trait abbreviation	Description	Ecological relevance	Reference
Adult	Snout-vent length	SVL	Distance from tip of snout to posterior margin of vent	Body size. Resource acquisition	Lescano et al., 2018; Campos et al., 2019; Wells, 2019; Pitogo et al., 2021
	Head length	HL	Distance from the posterior of the jaws to the tip of the snout	Resource use and acquisition	Fei et al., 2009a; Lescano et al., 2018; Pitogo et al., 2021
	Head width	HW	The maximum distance between the sides of the head		
	Eye diameter	ED	The horizontal distance from the anterior to posterior corner of the eye	Predation and anti-predator capabilities	Wilczynski and Capranica, 1984; Pereyra et al., 2016; Huang et al., 2019; Thomas et al., 2020
	Tympanum diameter	TD	Greatest horizontal width of the tympanum		
	Hand length	HAL	Distance from the base of the outer palmar tubercle to the tip of Finger III	Resource use. Locomotor ability and modes of locomotion	Fei et al., 2009a; Lescano et al., 2018; Pitogo et al., 2021
	Foot length	FL	Distance from the base of the inner metatarsal tubercle to the tip of Toe IV		
	Tibia length	TL	Distance from the outer surface of the flexed knee to the heel/tibiotarsal inflection		
	Arboreal	Arboreal	0: presence; 1: absence	Habitat use	Inger et al., 1986; McDiarmid and Altig, 1999; Fei et al., 2009a
	Fossorial	Fossorial	0: presence; 1: absence		
	Terrestrial	Terrestrial	0: presence; 1: absence		
	Aquatic	Aquatic	0: presence; 1: absence		
	Torrential	Torrential	0: presence; 1: absence		
Tadpole	Total length	TOL	Length from snout to end of tail	Body size. Resource acquisition	Altig and Johnston, 1989; McDiarmid and Altig, 1999; Baldo et al., 2014; Laudor et al., 2021
	Body length	BL	Length from snout to vent tube	Position in the water column and hydrodynamism	McDiarmid and Altig, 1999; Baldo et al., 2014; Sun et al., 2021
	Body width	BW	The maximum width on both sides of the body		
	Body height	BH	Maximum height between dorsal and ventral surfaces		
	Tail length	TL	Length from base to end of tail	Swimming ability and maneuverability	Jennings and Scott, 1993; McDiarmid and Altig, 1999; Sun et al., 2021
	Tail height	TH	Maximum height between upper and lower fins edges		
	Tail muscle width	TMW	The width of the tail muscles		
	Oral disc width	ODW	The maximum width of the oral disc	The ability to exploit food resources	Altig and Johnston, 1989; McDiarmid and Altig, 1999; Sun et al., 2021
	Lentic	Lentic	0: presence; 1: absence	Habitat use	McDiarmid and Altig, 1999; Fei et al., 2009a; Laudor et al., 2021
	Lotic	Lotic	0: presence; 1: absence		

Our compiled dataset includes functional trait data for 375 species. The completeness of the trait data is very high, morphological traits ranging from 68.7% to 100%, and information on microhabitat covered all adults and 76.5% of tadpoles (Figure 1—figure supplement 2). We performed data imputation considering phylogenetic relationships. The phylogenetic data we used came from previous studies (Xu et al., 2024; Song et al., 2025a), covering all Chinese anuran species. First, we calculated the phylogenetic distance matrix for the species based on the phylogenetic tree. Then, through principal coordinates analysis, we extracted the principal coordinates of the phylogenetic distance matrix, merged them with the morphological traits and microhabitat data. In total, we used 100 axes, which could account for 99.4% of the variances. Missing values were imputed using the R package missForest (Stekhoven, 2022), a random forest-based method proven highly accurate for high-dimensional data with complex, nonlinear interactions (Stekhoven and Bühlmann, 2012; Penone et al., 2014). We used its default configuration: maximum iterations were set to 10; initial fill values used the median for numerical variables and the mode for categorical variables; the random forest contained 100 decision trees, with a minimum leaf node sample size of 1, a minimum sample size for internal node splitting of 2. The algorithm iteratively refines predictions across variables until convergence or the maximum iteration limit is reached.

### Data analysis

Prior to analysis, we assessed multicollinearity among all trait variables using variance inflation factors (VIFs). The VIF for each variable was below 5, indicating acceptable levels of collinearity (Supplementary file 1B). We employed phylogenetic generalized least squares (PGLS) models to examine associations between species traits and threat categories. To account for non-independence due to shared evolutionary history, we incorporated a phylogenetic covariance matrix derived from the species tree. Following the method of Swenson, 2014, and Revell and Harmon, 2022, models were fitted in R using the gls function from the *nlme* package (Pinheiro et al., 2025), under both Brownian motion (BM) and Ornstein-Uhlenbeck (OU) models of trait evolution. To assess the impact of phylogenetic structure on model performance, we also fitted non-phylogenetic generalized least squares (GLS) models. Model performance was compared using the small-sample corrected Akaike information criterion (AICc), calculated with the MuMIn package (Bartoń, 2023).

To further ensure the robustness of our model results, we also conducted a multi-model inference analysis using the dredge function in the MuMIn package (Bartoń, 2023) based on the best-fitting evolutionary model and calculated trait importance. By comparing variable importance rankings and the full models between the adult and larval stages, we further assessed how traits from different life stages differ in predicting extinction risk. We also repeated the analysis based on the categories of the IUCN Red List.

Additionally, to test if body size of adults and tadpoles were correlated, we examined the correlation between adult snout-vent length and tadpole total length. To correct for phylogenetic non-independence in correlation estimates, we used the corphylo function from the ape package to compute phylogenetically informed Pearson correlations (Paradis and Schliep, 2019). We first assessed this relationship across all species, then repeated the analysis within families containing more than five species.

All data compilation, analysis, and visualization were performed in R version 4.4.1 (R Development Core Team, 2024). Original files and code are accessible at figshare (https://doi.org/10.6084/m9.figshare.31149463).

### Conclusion

This study demonstrates that extinction risk in Chinese anurans is shaped by functional traits across multiple life stages. On one hand, stage-specific traits reveal unique combinations of threats faced at different life stages. On the other hand, body size, as a cross-stage common trait, is associated with higher extinction risk in both stages. These findings emphasize that focusing solely on the adult stage will underestimate the overall extinction risks of species, limit our understanding of the threats species face, and reduce the effectiveness of biodiversity conservation measures. Based on our results, we recommend incorporating information from different life history stages when assessing species threat status. Although this study is based on anuran species, stage-specific vulnerability likely has broad applicability. Given the large amounts of animal species that have complex life histories, we suggest integrating information across all life stages to build more effective frameworks for global biodiversity conservation.

## Data Availability

The raw data and code for this study have been deposited and made publicly available at https://doi.org/10.6084/m9.figshare.31149463. The following dataset was generated: SongYF WangYL YuanZY ZhouWW LiQQ 2026Conservation Blind Spot: The Critical Role of Larval Stage in Assessing Extinction Risk-Code and original datafigshare10.6084/m9.figshare.31149463

## Additional files

Supplementary file 1. Regression analysis (A) and variance inflation factor (B) results for adult and tadpole traits in relation to extinction risk.

MDAR checklist

## References

[bib1] Altig R, Johnston GF (1989). Guilds of anuran larvae: relationships among developmental modes, morphologies, and habitats. Herpetological Monographs.

[bib2] (2026). The database of Chinese amphibians. http://www.amphibiachina.org/.

[bib3] Baldo D, Candioti FV, Haad B, Kolenc F, Borteiro C, Pereyra MO, Zank C, Colombo P, Bornschein MR, Sisa FN, Brusquetti F, Conte CE, Nogueira-Costa P, Almeida-Santos P, Pie MR (2014). Comparative morphology of pond, stream and phytotelm-dwelling tadpoles of the South American Redbelly Toads (Anura: Bufonidae: *Melanophryniscus* ). Biological Journal of the Linnean Society.

[bib4] Bartoń K (2023). R Package Version 1.47.5.

[bib5] Biek R, Funk WC, Maxell BA, Mills LS (2002). What is missing in amphibian decline research: insights from ecological sensitivity analysis. Conservation Biology.

[bib6] Calhoun AJK, Jansujwicz JS, Bell KP, Hunter ML (2014). Improving management of small natural features on private lands by negotiating the science-policy boundary for Maine vernal pools. PNAS.

[bib7] Campos FS, Lourenço-De-Moraes R, Rudoy A, Rödder D, Llorente GA, Solé M (2019). Ecological trait evolution in amphibian phylogenetic relationships. Ethology Ecology & Evolution.

[bib8] Caorsi V, Guerra V, Furtado R, Llusia D, Miron LR, Borges-Martins M, Both C, Narins PM, Meenderink SWF, Márquez R (2019). Anthropogenic substrate-borne vibrations impact anuran calling. Scientific Reports.

[bib9] Cardillo M (2021). Clarifying the relationship between body size and extinction risk in amphibians by complete mapping of model space. Proceedings. Biological Sciences.

[bib10] Chan HK, Shoemaker KT, Karraker NE (2014). Demography of Quasipaa frogs in China reveals high vulnerability to widespread harvest pressure. Biological Conservation.

[bib11] Chen C, Chen C, Wang Y (2019). Ecological correlates of extinction risk in Chinese amphibians. Diversity and Distributions.

[bib12] Chichorro F, Juslén A, Cardoso P (2019). A review of the relation between species traits and extinction risk. Biological Conservation.

[bib13] Chichorro F, Urbano F, Teixeira D, Väre H, Pinto T, Brummitt N, He X, Hochkirch A, Hyvönen J, Kaila L, Juslén A, Cardoso P (2022). Trait-based prediction of extinction risk across terrestrial taxa. Biological Conservation.

[bib14] Cooper N, Bielby J, Thomas GH, Purvis A (2008). Macroecology and extinction risk correlates of frogs. Global Ecology and Biogeography.

[bib15] Cowie RH, Bouchet P, Fontaine B (2022). The Sixth Mass Extinction: fact, fiction or speculation?. Biological Reviews of the Cambridge Philosophical Society.

[bib16] Damuth J (1981). Population density and body size in mammals. Nature.

[bib17] Faria L, Pie M, Salles F, Soares E (2021). The Haeckelian shortfall or the tale of the missing semaphoronts. Journal of Zoological Systematics and Evolutionary Research.

[bib18] Fei L, Hu S, Ye C (2009a). Fauna Sinica Amphibia. Vol.2. Anura.

[bib19] Fei L, Hu S, Ye C (2009b). Fauna Sinica Amphibia. Vol.3. Anura Ranidae.

[bib20] Fei L (2020). Atlas of Amphibians in China.

[bib21] Frost DR (2026). Amphibian species of the world: an online reference. Version 6.2. https://amphibiansoftheworld.amnh.org/index.php.

[bib22] Goedert D, Calsbeek R (2019). Experimental evidence that metamorphosis alleviates genomic conflict. The American Naturalist.

[bib23] González-del-Pliego P, Freckleton RP, Edwards DP, Koo MS, Scheffers BR, Pyron RA, Jetz W (2019). Phylogenetic and trait-based prediction of extinction risk for data-deficient amphibians. Current Biology.

[bib24] Grenat PR, Pollo FE, Ferrero MA, Martino AL (2019). Differential and additive effects of natural biotic and anthropogenic noise on call properties of *Odontophrynus americanus* (Anura, Odontophryinidae): Implications for the conservation of anurans inhabiting noisy environments. Ecological Indicators.

[bib25] Grenat P, Ferrero M, Baraquet M, Pollo F, Otero M, Salinas Z, Salas N, Martino A (2024). Changes in call properties of *Boana pulchella* (Anura, Hylidae) in response to different noise conditions. Current Zoology.

[bib26] Haas A, Das I, Hertwig ST (2022). A Guide to the Tadpoles of Borneo.

[bib27] Hamer AJ, Parris KM (2011). Local and landscape determinants of amphibian communities in urban ponds. Ecological Applications.

[bib28] Holzer KA (2014). Amphibian use of constructed and remnant wetlands in an urban landscape. Urban Ecosystems.

[bib29] Huang CH, Zhong MJ, Liao WB, Kotrschal A (2019). Investigating the role of body size, ecology, and behavior in anuran eye size evolution. Evolutionary Ecology.

[bib30] Huang N, Sun X, Song Y, Yuan Z, Zhou W (2023). Amphibian traits database: A global database on morphological traits of amphibians. Global Ecology and Biogeography.

[bib31] Inger RF, Szarski H, Kollros JJ, Duellman WE, Trueb L (1986). Biology of amphibians. Copeia.

[bib32] (2025). The IUCN Red List of Threatened Species Version 2025-2. https://www.iucnredlist.org.

[bib33] Jennings RD, Scott NJ (1993). Ecologically correlated morphological variation in tadpoles of the leopard frog, *Rana chiricahuensis*. Journal of Herpetology.

[bib34] Keane A, Brooke M, Mcgowan PJK (2005). Correlates of extinction risk and hunting pressure in gamebirds (Galliformes). Biological Conservation.

[bib35] Keck F, Peller T, Alther R, Barouillet C, Blackman R, Capo E, Chonova T, Couton M, Fehlinger L, Kirschner D, Knüsel M, Muneret L, Oester R, Tapolczai K, Zhang H, Altermatt F (2025). The global human impact on biodiversity. Nature.

[bib36] Laudor J, Schulze A, Veith M, Viertel B, Elle O, Lötters S (2021). Morphology of lentic and lotic tadpoles from Madagascar. BMC Zoology.

[bib37] Lescano JN, Miloch D, Leynaud GC (2018). Functional traits reveal environmental constraints on amphibian community assembly in a subtropical dry forest. Austral Ecology.

[bib38] Liu X, Li S, Feng Y, Chen X, Ma Y, Xiao H, Zhao Y, Liu S, Zheng G, Yang X, Wu F, Xie J (2024). Traditional knowledge of animal-derived medicines used by Gelao community in Northern Guizhou, China. Journal of Ethnobiology and Ethnomedicine.

[bib39] Luo C, Zhao W, Liu S, Luo M, Fan T, Zhao Y, Ren Y, Wu F, Xie J (2023). Animal-and mineral-based medicines in Gansu-Ningxia-inner Mongolia region, P.R. China: a cross-cultural ethnobiological assessment. Frontiers in Pharmacology.

[bib40] McDiarmid RW, Altig R (1999). Tadpoles: The Biology of Anuran Larvae.

[bib41] Moor H, Bergamini A, Vorburger C (2022). Bending the curve: Simple but massive conservation action leads to landscape-scale recovery of amphibians. PNAS.

[bib42] Moran NA (1994). Adaptation and constraint in the complex life cycles of animals. Annual Review of Ecology and Systematics.

[bib43] Ning L, Yu S, Wang P, Li R, Zhu D, Zhang J (2025). Climate change risk to giant panda populations: insights from changes in both habitat area and bioclimatic velocity. Global Change Biology.

[bib44] Nolan N, Hayward MW, Klop-Toker K, Mahony M, Lemckert F, Callen A (2023). Complex organisms must deal with complex threats: how does amphibian conservation deal with biphasic life cycles?. Animals.

[bib45] Nolte D, Boutaud E, Kotze DJ, Schuldt A, Assmann T (2019). Habitat specialization, distribution range size and body size drive extinction risk in carabid beetles. Biodiversity and Conservation.

[bib46] Nori J, Baldo D, Pereyra M, Grosjean S, dos Santos Dias PH, Müller H, Cordier M, Huais PY, Tomba A, Vera Candioti F (2025). Global key areas for anuran tadpole discovery. Biological Journal of the Linnean Society.

[bib47] Owens IP, Bennett PM (2000). Ecological basis of extinction risk in birds: habitat loss versus human persecution and introduced predators. PNAS.

[bib48] Paluh DJ, Stanley EL, Blackburn DC (2020). Evolution of hyperossification expands skull diversity in frogs. PNAS.

[bib49] Paradis E, Schliep K (2019). ape 5.0: an environment for modern phylogenetics and evolutionary analyses in R. Bioinformatics.

[bib50] Penone C, Davidson AD, Shoemaker KT, Di Marco M, Rondinini C, Brooks TM, Young BE, Graham CH, Costa GC (2014). Imputation of missing data in life‐history trait datasets: which approach performs the best?. Methods in Ecology and Evolution.

[bib51] Pereyra MO, Womack MC, Barrionuevo JS, Blotto BL, Baldo D, Targino M, Ospina-Sarria JJ, Guayasamin JM, Coloma LA, Hoke KL, Grant T, Faivovich J (2016). The complex evolutionary history of the tympanic middle ear in frogs and toads (Anura). Scientific Reports.

[bib52] Petrovan SO, Schmidt BR (2019). Neglected juveniles; a call for integrating all amphibian life stages in assessments of mitigation success (and how to do it). Biological Conservation.

[bib53] Phillips PC (1998). Genetic constraints at the metamorphic boundary: Morphological development in the wood frog, *Rana sylvatica*. Journal of Evolutionary Biology.

[bib54] Phung TX, Nascimento JCS, Novarro AJ, Wiens JJ (2020). Correlated and decoupled evolution of adult and larval body size in frogs. Proceedings. Biological Sciences.

[bib55] Pinheiro J, Bates D (2025). Nlme.

[bib56] Pitogo KME, Saavedra AJL, Aurellado MEB, de Guia APO, Afuang LE (2021). Functional traits and environment drive montane amphibian distribution in the southern Philippines. Biodiversity and Conservation.

[bib57] Purvis A, Gittleman JL, Cowlishaw G, Mace GM (2000). Predicting extinction risk in declining species. Proceedings of the Royal Society of London. Series B.

[bib58] (2024). http://www.r-project.org.

[bib59] Revell LJ, Harmon LJ (2022). Phylogenetic Comparative Methods in R.

[bib60] Roehrdanz PR (2026). Beyond species ranges: How functional diversity and integrated life history can inform conservation priorities. PNAS.

[bib61] Ruland F, Jeschke JM (2017). Threat-dependent traits of endangered frogs. Biological Conservation.

[bib62] Schwartz JJ, Buchanan BW, Gerhardt HC (2001). Female mate choice in the gray treefrog (Hyla versicolor) in three experimental environments. Behavioral Ecology and Sociobiology.

[bib63] Seibold S, Brandl R, Buse J, Hothorn T, Schmidl J, Thorn S, Müller J (2015). Association of extinction risk of saproxylic beetles with ecological degradation of forests in Europe. Conservation Biology.

[bib64] Sherratt E, Vidal-García M, Anstis M, Keogh JS (2017). Adult frogs and tadpoles have different macroevolutionary patterns across the Australian continent. Nature Ecology & Evolution.

[bib65] Song Y-F, Liu X-L, Liu J-J, Li Q-Q, Li X-Q, Lu W-Z, Zhou W-W, Yuan Z-Y (2025a). Biodiversity conservation requires consideration of different life history stages. PNAS.

[bib66] Song Y, Tian R, Wang Y, Wei P, Zhang T, Luo S, Yuan Z, Zhou W (2025b). TadpoleTraits: a global database on morphological traits of tadpoles of Anura. Global Ecology and Biogeography.

[bib67] Stekhoven DJ, Bühlmann P (2012). MissForest--non-parametric missing value imputation for mixed-type data. Bioinformatics.

[bib68] Stekhoven DJ (2022). R Package Version15.

[bib69] Sun Z, Zhao C, Zhu W, Zhu W, Feng J, Su S, Zhao T (2021). Microhabitat features determine the tadpole diversity in mountainous streams. Ecological Indicators.

[bib70] Swenson NG (2014). Functional and Phylogenetic Ecology in R.

[bib71] Terzopoulou S, Rigal F, Whittaker RJ (2015). Drivers of extinction: the case of Azorean beetles. Biology Letters.

[bib72] (2023). Red List of China’s Biodiversity Vertebrate Volume (2020). https://www.mee.gov.cn/xxgk2018/xxgk/xxgk01/202305/W020230522536559098623.pdf.

[bib73] Thomas KN, Gower DJ, Bell RC, Fujita MK, Schott RK, Streicher JW (2020). Eye size and investment in frogs and toads correlate with adult habitat, activity pattern and breeding ecology. Proceedings of the Royal Society B.

[bib74] Tian YB, Xia MM, Shao C (2011). Advances in germplasm resource research of *Hoplobatrachus chinensis*. Journal of Zhejiang Normal University.

[bib75] Tobias JA, Sheard C, Pigot AL, Devenish AJM, Yang J, Sayol F, Neate-Clegg MHC, Alioravainen N, Weeks TL, Barber RA, Walkden PA, MacGregor HEA, Jones SEI, Vincent C, Phillips AG, Marples NM, Montaño-Centellas FA, Leandro-Silva V, Claramunt S, Darski B, Freeman BG, Bregman TP, Cooney CR, Hughes EC, Capp EJR, Varley ZK, Friedman NR, Korntheuer H, Corrales-Vargas A, Trisos CH, Weeks BC, Hanz DM, Töpfer T, Bravo GA, Remeš V, Nowak L, Carneiro LS, Moncada R AJ, Matysioková B, Baldassarre DT, Martínez-Salinas A, Wolfe JD, Chapman PM, Daly BG, Sorensen MC, Neu A, Ford MA, Mayhew RJ, Fabio Silveira L, Kelly DJ, Annorbah NND, Pollock HS, Grabowska-Zhang AM, McEntee JP, Carlos T Gonzalez J, Meneses CG, Muñoz MC, Powell LL, Jamie GA, Matthews TJ, Johnson O, Brito GRR, Zyskowski K, Crates R, Harvey MG, Jurado Zevallos M, Hosner PA, Bradfer-Lawrence T, Maley JM, Stiles FG, Lima HS, Provost KL, Chibesa M, Mashao M, Howard JT, Mlamba E, Chua MAH, Li B, Gómez MI, García NC, Päckert M, Fuchs J, Ali JR, Derryberry EP, Carlson ML, Urriza RC, Brzeski KE, Prawiradilaga DM, Rayner MJ, Miller ET, Bowie RCK, Lafontaine R-M, Scofield RP, Lou Y, Somarathna L, Lepage D, Illif M, Neuschulz EL, Templin M, Dehling DM, Cooper JC, Pauwels OSG, Analuddin K, Fjeldså J, Seddon N, Sweet PR, DeClerck FAJ, Naka LN, Brawn JD, Aleixo A, Böhning-Gaese K, Rahbek C, Fritz SA, Thomas GH, Schleuning M (2022). AVONET: morphological, ecological and geographical data for all birds. Ecology Letters.

[bib76] Tomiya S (2013). Body size and extinction risk in terrestrial mammals above the species level. The American Naturalist.

[bib77] Uetz P, Freed P, Aguilar R (2025). The reptile database. http://www.reptile-database.org.

[bib78] Verde Arregoitia LD (2016). Biases, gaps, and opportunities in mammalian extinction risk research. Mammal Review.

[bib79] Vilela B, Villalobos F, Rodríguez MÁ, Terribile LC (2014). Body size, extinction risk and knowledge bias in New World snakes. PLOS ONE.

[bib80] Violle C, Navas ML, Vile D (2007). Let the concept of trait be functional. Oikos.

[bib81] Vredenburg VT (2004). Reversing introduced species effects: Experimental removal of introduced fish leads to rapid recovery of a declining frog. PNAS.

[bib82] Wei P, Luo X, Pie MR, Sucharitakul P, Zhou W, Yuan Z (2026). Global diversity patterns in anurans are determined by terrestrial and arboreal species. Integrative Zoology.

[bib83] Wells KD (2019). The Ecology and Behavior of Amphibians.

[bib84] Werner E, Ebenman B, Persson L (1988). Size-Structured Populations: Ecology and Evolution.

[bib85] Wilczynski W, Capranica RR (1984). The auditory system of anuran amphibians. Progress in Neurobiology.

[bib86] Wollenberg Valero KC, Garcia-Porta J, Rodríguez A, Arias M, Shah A, Randrianiaina RD, Brown JL, Glaw F, Amat F, Künzel S, Metzler D, Isokpehi RD, Vences M (2017). Transcriptomic and macroevolutionary evidence for phenotypic uncoupling between frog life history phases. Nature Communications.

[bib87] Womersley FC, Sousa LL, Humphries NE, Abrantes K, Araujo G, Bach SS, Barnett A, Berumen ML, Lion SB, Braun CD, Clingham E, Cochran JEM, de la Parra R, Diamant S, Dove ADM, Duarte CM, Dudgeon CL, Erdmann MV, Espinoza E, Ferreira LC, Fitzpatrick R, Cano JG, Green JR, Guzman HM, Hardenstine R, Hasan A, Hazin FHV, Hearn AR, Hueter RE, Jaidah MY, Labaja J, Ladino F, Macena BCL, Meekan MG, Morris JJ, Norman BM, Peñaherrera-Palma CR, Pierce SJ, Quintero LM, Ramírez-Macías D, Reynolds SD, Robinson DP, Rohner CA, Rowat DRL, Sequeira AMM, Sheaves M, Shivji MS, Sianipar AB, Skomal GB, Soler G, Syakurachman I, Thorrold SR, Thums M, Tyminski JP, Webb DH, Wetherbee BM, Queiroz N, Sims DW (2024). Climate-driven global redistribution of an ocean giant predicts increased threat from shipping. Nature Climate Change.

[bib88] Xu W, Wu Y-H, Zhou W-W, Chen H-M, Zhang B-L, Chen J-M, Xu W, Rao D-Q, Zhao H, Yan F, Yuan Z, Jiang K, Jin J-Q, Hou M, Zou D, Wang L-J, Zheng Y, Li J-T, Jiang J, Zeng X-M, Chen Y, Liao Z-Y, Li C, Li X-Y, Gao W, Wang K, Zhang D-R, Lu C, Yin T, Ding Z, Zhao G-G, Chai J, Zhao W-G, Zhang Y-P, Wiens JJ, Che J (2024). Hidden hotspots of amphibian biodiversity in China. PNAS.

[bib89] Zhao LX, Li RQ, Xiao ZJ (2014). Research status on the development and utilization of *Rana dybowskii*. Journal of Veterinary Science and Technology Information.

[bib90] Zhao L, Deng K, Wang T, Guo R, Cui J, Wang J (2025a). Anuran communities increase aggregations of conspecific calls in response to aircraft noise. Current Zoology.

[bib91] Zhao Y, Liu J, Wang F, Zhang Y, Ma Z (2025b). Targeted conservation actions are required to save species from extinction. Biological Conservation.

