# Supplementary material for: Stage-specific threats reveal the inadequacy of adult-centered conservation
Source: eLife. 2026 Sep 7;15:RP110823. doi: 10.7554/eLife.110823 (PMC13549618; doi:10.7554/eLife.110823)
Supplement: Supplementary file 2 [file elife-110823-supp1.docx]

**Supplementary File 1**

Table A. Regression analysis results of extinction risk for adult and tadpole stages (IUCN). For abbreviations of traits, please refer to Table 1. BM, Brownian motion; OU, Ornstein–Uhlenbeck; GLS, generalized least squares; PGLS, phylogenetic generalized least squares; AICc, Akaike Information Criterion corrected for small sample size.

|  | **Model type** | **AICc** | **Model** |
| --- | --- | --- | --- |
| Adult | PGLS (BM) | 1134.92 | / |
|  | PGLS (OU) | 885.32 | IUCN extinction risk ~ 0.19 SVL + 4.04 HL + 0.60 HW + 5.76 ED - 10.81 TD*** + 1.16 HAL - 1.26 FL + 0.44 TL - 0.17 Arboreal - 0.15 Fossorial + 0.21 Terrestrial - 0.16 Aquatic + 0.0004 Torrential |
|  | GLS (no-phylogenetic) | 885.65 | / |
| Tadpole | PGLS (BM) | 1133.05 | / |
|  | PGLS (OU) | 873.81 | IUCN extinction risk ~ 0.53 TOL* + 4.04 BL - 2.57 BW - 0.73 BH + 0.37 TL - 0.68 TH - 3.68 TMW + 2.99 MW - 0.57 Lentic - 0.05 Lotic |
|  | GLS (no-phylogenetic) | 874.40 | / |

Table B. The results of variance inflation factors for adult and tadpole traits. For abbreviations of traits, please refer to Table 3.

| **Life stage** | **Trait** | **VIF value** |
| --- | --- | --- |
| Adult | SVL | 1.5 |
|  | HL | 2.0 |
|  | HW | 2.1 |
|  | ED | 1.5 |
|  | TD | 1.4 |
|  | HAL | 1.1 |
|  | FL | 1.5 |
|  | TL | 1.6 |
| Tadpole | TOL | 1.2 |
|  | SVL | 2.9 |
|  | BW | 3.6 |
|  | BH | 4.5 |
|  | TL | 1.9 |
|  | TH | 2.2 |
|  | TMW | 1.3 |
|  | MW | 1.3 |
