# Supplementary material for: Stage-specific threats reveal the inadequacy of adult-centered conservation
Source: eLife. 2026 Sep 7;15:RP110823. doi: 10.7554/eLife.110823 (PMC13549618; doi:10.7554/eLife.110823)
Supplement: Peer review history

# Editor report: eLife Assessment

**DOI:** 10.7554/eLife.110823.3.sa0

**Authors:** Bernhard Schmid

**Affiliations:** University of Zurich Switzerland

**Keywords:** Solid, Valuable

This **valuable** study analyses correlations between traits of Chinese frog species and their Red List status and finds differences between adults and larvae. Of broad relevance, this **solid** study makes the statement to consider different life-cycle stages when assessing species extinction risks, although many conclusions are based on limited data and thus offer hypotheses rather than direct conservation advice.

# Referee report: Reviewer #1 (Public review)

**DOI:** 10.7554/eLife.110823.3.sa1

[Editors' note: this version has been assessed by the Reviewing Editor without further input from the original reviewers. The authors have addressed the major comments raised in the previous round of reviews, yet some inherent issues necessarily remain unresolved.]

The manuscript shows that different traits of adults and larvae correlate with Red List status. The authors argue that this shows a big gap in the conservation of amphibians and that the traits of all life stages should be taken into account in amphibian conservation. Specifically, amphibian conservation should do more for the habitats where the larvae live.

The manuscript is well written and easy to understand. The methods are sound.

# Referee report: Reviewer #2 (Public review)

**DOI:** 10.7554/eLife.110823.3.sa2

Summary:

In this study, the authors tried to examine whether there are differences in the association between functional traits and extinction risk in adult and tadpole stages in Chinese anurans.

Strengths:

Overall, I think the basic idea of the study is interesting and important. It can be applied to other taxa with complex life cycles throughout the animal kingdom.

Original weaknesses:

I do not think the authors achieve their aims, as the results only partially support their conclusions. The study has several drawbacks that need to be clarified or revised, including the unclear threat categories for tadpoles, model selection and model averaging, the potential problem of AIC, and the omission of other important species traits.

# Author comment: Author response

**DOI:** 10.7554/eLife.110823.3.sa3

**Authors:** Yan-Fang Song, Yong-Le Wang, Qing-Qing Li, Zhi-Yong Yuan, Wei-Wei Zhou

**Affiliations:** Southwest Forestry University Kunming China; Lanzhou University Lanzhou China; Southwest Forestry University Kunming China; Southwest University Chongqing China; Lanzhou University Lanzhou China

The following is the authors’ response to the original reviews.

**eLife Assessment**

This valuable study analyses correlations between traits of Chinese frog species and their Red List status, finding differences between adults and larvae and thus pointing to the importance of considering different life-cycle stages in this and possibly other animal groups when assessing species extinction risks. The current study is, however, incomplete because of unclear threat categories for tadpoles, the omission of other key species traits, and insufficient statistical analysis.

Thank you very much. We have revised the manuscript according to the reviewers' comments. The parts highlighted in red in the manuscript are the revised portions.

**Public Reviews:**

**Reviewer #1 (Public review):**

The manuscript shows that different traits of adults and larvae correlate with Red List status. The authors argue that this shows a big gap in the conservation of amphibians and that the traits of all life stages should be taken into account in amphibian conservation. Specifically, amphibian conservation should do more for the habitats where the larvae live.

The manuscript is well written and easy to understand. The methods are sound.

While the study will make an interesting contribution to conservation science, there are many things that I disagree with.

(1) I don't think that amphibian larvae and their requirements are a "blind spot" as the title suggests. When reading the manuscript, I didn't learn how conservation practice should change in response to the results.

Thank you very much for your suggestions. The description of the 'blind spot' was inappropriate, and we have revised it. Investigating the relationship between life history traits and threat status can help us understand which species are more vulnerable to extinction. Furthermore, we can predict the potential threat severity of species that have not yet been assessed. Because we still lack knowledge about the biodiversity of many taxonomic groups. For example, as of early 2024, over 34% of Chinese anuran species have been described in the last ten years, and 100 - 200 new species are still being discovered globally each year. Under these circumstances, given the current investment in biodiversity conservation, it is nearly impossible to assess the threat status of every species and develop conservation strategies. Therefore, predicting the threat status of species is very important for biodiversity conservation, as it will provide support for the subsequent formulation of specific conservation policies. Among the already described animals species, most have complex life history cycles. Moreover, species face threats not only at the adult stage; those with certain traits at other life stages may also be vulnerable to threats. For example, our study takes amphibians as an example and shows that groups with larger body sizes at the tadpole stage may face more serious threats.

(2) I wonder whether the relationship between species traits and extinction risk is of great importance for conservation. If a species is Data Deficient on the IUCN Red List, then species traits could be used to predict its Red List category. However, for other conservation projects, I don't see how this would work. How would traits be linked to captive breeding, conservation translocation, pond construction or habitat management in general? In some cases, I can envision a link between species traits and pond hydroperiod.

Thank you very much for your suggestions. Understanding the relationship between traits and threat status is of great importance for the conservation policies and the allocation of conservation resources, especially when conservation resources are insufficient. As mentioned earlier, the current conservation resources are insufficient to support us in surveying and assessing every Data Deficient (DD) species, not to mention the large number of new species being discovered each year. By predicting threat status, we can identify which groups or species should be prioritized for research, such as population size and distribution range surveys, so that specific conservation strategies can subsequently be developed.

(3) Species traits are body size and morphological traits. That makes sense. However, one of the species traits was microhabitat. I find it far-fetched to call habitat a species trait. This is standard habitat ecology. It is well known that habitats matter and that different habitat types face different threats, and consequently, the species that live in those habitats. Furthermore, habitat and morphology may be confounded. For example, tadpoles in lentic and lotic habitats have very different morphologies. So is it habitat or morphology?

Thank you very much for your suggestions. The type of habitat in which a species lives affects the threats it faces. In many studies on the relationship between extinction risk and traits, microhabitat or habitat type is widely used as a predictive variable. For example, in studies on Squamata, whether a species is distributed on islands or peninsulas has also been included as a trait. Following your suggestion, we have revised the sentences to refer to 'morphological traits and microhabitat information'. Many morphological traits of species are related to habitat selection, but not all traits associated with habitat selection have been measured or have sufficient data. Therefore, it is necessary to include microhabitat type as an independent variable. Additionally, we calculated the Variance Inflation Factor (VIF) prior to the regression analysis to ensure that the analysis was not affected by multicollinearity.

(4) I don't know how the threat status of Chinese amphibians is determined. IUCN has multiple reasons why a species can be Red Listed. One reason is range size, and another reason is population decline. Personally, I don't think they should be pooled in an analysis because they are fundamentally different reasons why a species has a high extinction risk. A reduction in population size of greater than 30% in 10 years or 3 generations is not the same thing as a small distribution range. Another issue is that IUCN developed the Green Status of species. The Green Status shows that even a species which is LC on the Red List may be significantly depleted.

Thank you very much for your valuable suggestions. The assessment method of the China Biodiversity Red List is the same as that of the IUCN Red List, both of which are based on population size and area of distribution. We fully agree with your point that analyses should be conducted according to specific threat types. Unfortunately, the full report of the latest version of the China Biodiversity Red List, released in 2023, has still not been published. Therefore, we were unable to perform the relevant analyses.

(5) The species traits in Table 1 are mostly functional/morphological and body size related (and microhabitat). While there may be correlations between traits and Red List status, it is unknown whether this is correlation or causation. In addition, it is difficult to know the conservation interventions that may be necessary now that we know that relative head with and Red List status are correlated.

Thank you for pointing out the important distinction between correlation and causation. Your comment is very insightful, and we have revised our manuscript to further clarify the scope and limitations of our study. The aim of our study is to identify which traits show statistical associations with extinction risk, thereby providing testable hypotheses for future research. We acknowledge that the mechanisms underlying the associations between certain morphological traits (e.g., head length, tympanum diameter) and extinction risk remain unclear, and these findings cannot yet be directly translated into well-established management measures. Nevertheless, the value of our study lies precisely in generating hypotheses about traits that warrant prioritized investigation of their causal mechanisms, as well as offering clues for the initial allocation of conservation resources. Following your suggestion, we have discussed the limitations of the study in the Discussion section of the manuscript.

(6) In the discussion, the authors explain why body size and other traits may affect extinction risk and whether there is a causal relationship. I agree that body size may have a direct effect because larger species are harvested more frequently (it was interesting to learn that tadpoles are harvested as well). However, as macroecological studies show, smaller species often have larger populations than larger species. Abundance may matter.

Thank you very much for your suggestion. Following your advice, we have revised the discussion section regarding body size.

(7) I found it much harder to understand why relative head length and tympanum size correlated with Red List status. I wasn't convinced by the arguments in the discussion. Typanum size may be related to hearing and anthropogenic noise. Several studies are cited which show that frogs alter their calling behaviour in response to noise. Crucially, however, they describe changes in behaviour or properties of the advertisement call, yet none show that noise has effects on population viability. If some anthropogenic stressor affects individuals, then this does not mean that it will cause a population decline. When IUCN published the second global amphibian assessment, did they list noise as a major threat to amphibians?

We appreciate your insightful comments and fully agree with your assessment. Indeed, the hypothesis that noise threatened anuran amphibians lacks direct evidence. While relevant studies indicate that anthropogenic noise causes auditory masking in anurans and reduces individual reproductive success, the IUCN has not listed noise as a primary threat to amphibians. Although acoustic communication is vital for amphibian reproduction and is susceptible to noise interference, there is currently no definitive evidence proving that noise extensively impacts amphibian survival. Therefore, in the revised manuscript, we retained it as a hypothesis to be tested and explicitly clarified that current evidence is limited to behavioral changes. Regarding the correlation with relative head length, we acknowledge that the underlying mechanism remains unclear; it may stem from phylogenetic signal residuals or unidentified ecological factors (such as diet or locomotor ability). In the Discussion, we revised this part as a correlation requiring further investigation.

(8) There are statements that the tadpole stage is the most important stage: "a critical period for amphibian survival" (line 78-79). While there is high mortality in the tadpole stage, tadpole survival is rather unlikely to affect population survival. Many population models show this. See, for example, Biek et al. 2002 in Conservation Biology. Other papers have argued that the postmetamorphic juvenile stage is most important (Petrovan and Schmidt 2009 Biological Conservation).

We greatly appreciate your comment. We agree that the original statement was overly absolute. The most critical life stage for population persistence can differ across species, and many studies have shown that other stages may be more important. Accordingly, we have revised this sentence as you suggested.

(9) The authors repeatedly make the statement that amphibian conservation should focus more on the tadpole stage. I don't understand why this statement is made. For example, a major activity in amphibian conservation is the restoration and de novo construction of ponds (see Calhoun et al. 2014 PNAS, Moor et al. 2022 PNAS). Ponds are habitats for tadpoles. Others removed fish from amphibian breeding sites because fish prey on tadpoles (and adults; see Vredenburg 2004 PNAS). Semlitsch (2002 in Conservation Biology) argued that the management of pond hydroperiod is a critical element of amphibian recovery plans. Ponds should be temporary because this effectively removes predators that consume tadpoles. Clearly, the tadpole stage is not a neglected stage in amphibian conservation.

Thank you for pointing this out. The literature you cited (Calhoun et al., 2014; Moor et al., 2022; Vredenburg, 2004; Semlitsch, 2002) convincingly demonstrates that the tadpole stage has received a certain degree of attention in amphibian conservation practice. Our original statement was indeed problematic. What we intended to convey is that information on the tadpole stage needs to be integrated into conservation assessment frameworks and conservation planning. For example, many studies on the relationship between functional traits and threat extent have not included tadpole-related information. Compared with our knowledge of adult amphibians, we know far less about tadpoles, and for many species, information on the tadpole stage is entirely lacking. Therefore, we call for tadpoles to receive greater attention in future research relative to the current situation.

**Recommendations for the authors:**

**Reviewing Editor Comments:**

Conceptual problems:

(1) Many conservation measures for amphibians target larvae; thus, globally, this is not a blind spot. If this is different in China, it would be important to point this out.

We thank the reviewer for the thoughtful comment. We recognize that the tadpole stage has indeed received attention in amphibian conservation practice, and our original statement was therefore imprecise. Our intended argument was that tadpole-stage information should be integrated into conservation assessment frameworks and conservation planning. For instance, many studies examining the relationships between functional traits and threat extent have failed to include data on tadpoles. Our understanding of tadpoles remains far more limited than that of adult amphibians, and for a large number of species, no information on the tadpole stage is available. Consequently, we advocate for substantially greater research attention to tadpoles than they currently receive. We have revised the text accordingly.

(2) While traits may be used to predict Red-List status, it is not clear how they could inform conservation measures. This should be discussed.

Thank you for your comment. The aim of our study is to identify which traits show statistical associations with extinction risk, thereby providing testable hypotheses for future research. We acknowledge that the mechanisms underlying the associations between certain morphological traits (e.g., head length, tympanum diameter) and extinction risk remain unclear, and these findings cannot yet be directly translated into well-established management measures. Nevertheless, the value of our study lies precisely in generating hypotheses about traits that warrant prioritized investigation of their causal mechanisms, as well as offering clues for the initial allocation of conservation resources. Following your suggestion, we have discussed the limitations of the study in the conclusion section of the manuscript.

(3) The Red-List categories may not be appropriate to link traits to extinction risk. It would be important to explain how these are defined for China and how this may affect the analysis (e.g. linking larval traits to larval extinction risks would be difficult if Red-List criteria do not consider larvae).

Thank you very much for your suggestions. The assessment method of the China Biodiversity Red List is the same as that of the IUCN Red List, both of which are based on population size and area of distribution. The assessment process is independent of species' morphological traits. Consequently, analyzing correlations between traits and Red List categories does not constitute circular reasoning or contain any inherent logical contradiction. On the contrary, it is precisely because the two are independent that statistically significant associations between traits and extinction risk can have predictive value and inform conservation actions. In the revised manuscript, we clarified the independence of Red List assessments and rephrase any potentially misleading wording (e.g., changing "threat category of tadpoles" to "threat category of the species (assessed based on adults)").

Methodological problems:

(4) Choice of traits. Are morphological traits sufficient (add e.g. fecundity)? Justify the use of habitat traits (also, if additional ones would be included: geographic and altitudinal ranges, habitat specificity).

Thank you for your suggestion. We fully agree that traits such as geographic range, elevational range, fecundity, and habitat specificity have important effects on extinction risk. The core objective of this study is to compare the stage-specific differences in the associations between extinction risk and morphological and microhabitat traits of adults versus tadpoles. Moreover, spatial traits such as geographic range are inherently highly correlated with the threat status of species, and including them might mask life-stage-specific signals. We will acknowledge this limitation in the discussion and identify the above-mentioned traits as important directions for future research.

(5) Model choice: models have high uncertainty, thus better use model averaging and AICc instead of AIC. Overall, the statistical analysis and model selection procedure are poorly described; only summary results are presented.

We greatly appreciate the reviewer's suggestion. Accordingly, we re-analyzed the data following your advice. In addition, the description of the methods has been supplemented.

(6) Caveats: the data only allow for correlational analysis; causation cannot be derived from observational data. Furthermore, with a limited number of species, the number of predictors should not be too large.

Thank you for your suggestion. Studying the relationship between traits and species threat status is important in conservation biology. Although such studies can only reveal statistical associations between traits and extinction risk rather than infer causality, they can generate hypotheses to facilitate future research. Additionally, this type of study can help predict the threat severity of unevaluated species, which is highly valuable for developing biodiversity conservation plans. In this study, 299 species were included in the analysis, and nine predictor variables (eight morphological traits plus one microhabitat type) were used. The ratio of sample size to number of variables was approximately 33:1, and variance inflation factor (VIF) tests indicated that multicollinearity was within an acceptable range (VIF < 5). Therefore, the risk of model overfitting is low. We will add this clarification in the revised manuscript.

**Reviewer #2 (Recommendations for the authors):**

(1) My first major concern is the species threat categories for tadpoles. The authors obtained the extinction risk data from the China Biodiversity Red List or IUCN. However, the assessment of threat categories, whether by the China Biodiversity Red List or IUCN, is based solely on adults. That means that the threat categories for both adults and tadpoles are the same, which can be seen in Figure 1. Since there is no specific assessment of threat categories for tadpoles, I have concerns about whether it is reasonable to relate species traits of tadpoles to the extinction risk for adults. I think it is one of the reasons why there is no study examining the association between functional traits and extinction risk in tadpole stages.

We thank the reviewer for raising this important point, as it addresses a key prerequisite issue. The Red List assessment evaluates species, not individual life stages. The threat categories of both the IUCN and China Biodiversity Red Lists are determined based on criteria such as population size and geographic range of the species. The assessment process is independent of species' morphological traits. Consequently, analyzing correlations between traits and Red List categories does not constitute circular reasoning or contain any inherent logical contradiction. On the contrary, statistically significant associations between traits and extinction risk can have predictive value and inform conservation actions. In the revised manuscript, we will explicitly clarify the independence of Red List assessments and rephrase any potentially misleading wording (e.g., changing "threat category of tadpoles" to "threat category of the species (assessed based on adults)").

(2) My second major concern is about the Data Analysis. The authors built and compared three types of models, i.e., PGLS_BM, PGLS_OU, and GLS_no_phylogeny. They claim that the OU-based PGLS model provided the best fit for both adult and tadpole datasets. Although the result seems reasonable, it is not clear how the OU-based PGLS model was obtained and what it exactly means. It seems to be a full model including all the predictor variables. However, since eight morphological traits and one microhabitat data of both adults and tadpoles were collected, there should be 29-1=511 candidate models. Unless the best model has an Akaike weight (wi) > 0.90 in all the OU-based PGLS models, it has substantial model selection uncertainty. If this is the case, the model average should be used, and weighted estimates of regression coefficients and unconditional standard errors that incorporate model selection uncertainty are better statistical methods (Burnham & Anderson, 2002).

Thank you very much for your suggestion. Species' traits are related to evolutionary relationships, with more closely related species tending to be more similar. In the original manuscript, the three models we compared (PGLS_BM, PGLS_OU, GLS_no_phylogeny) were intended to select the optimal evolutionary covariance structure. Since we were more interested in the differences between adults and tadpoles, after selecting the OU structure, we actually used a single full model that included all traits to estimate the regression coefficients for each factor. Following your advice, we have added a model averaging analysis and revised the manuscript accordingly.

(3) In addition, the Second-Order Information Criterion AICc, but not AIC, should be used for model selection. You have at least 9 variables (eight morphological traits and one microhabitat data) or 11/13 variables for the parameter estimates (Table 1). However, you have only 299 species included in the analysis (n = 299), which is relatively small compared to the number of variables (n/k << 40). Therefore, the AIC corrected for small sample size (AICc) should be used.

We greatly appreciate the reviewer's suggestion. Accordingly, we re-analyzed the data following your advice.

(4) Previous studies found that amphibian species with large body size, restricted geographic and elevational ranges, low fecundity or high habitat specificity are frequently predicted to have higher extinction risk (Cooper et al., 2008; Sodhi et al., 2008; Botts et al., 2013; Lips et al., 2003; Murray & Hose, 2005). The authors only included morphological traits and one microhabitat data point in the analyses. I wonder whether they can collect more trait data associated with extinction risk, such as geographic and elevational ranges, fecundity traits, or diet/habitat specificity, so as to gain more insight into the study.

Thank you for your suggestion. We fully agree that traits such as geographic range, elevational range, fecundity, and habitat specificity have important effects on extinction risk. The object of this study is to compare the stage-specific differences in the associations between extinction risk and morphological and microhabitat traits of adults versus tadpoles. Moreover, spatial traits such as geographic range are inherently highly correlated with the threat status of species, and including them might mask life-stage-specific signals. In the Methods, we acknowledge this limitation and identify the above-mentioned traits as important directions for future research.
